# Editorial: Machine learning in research on violence and general offending

**DOI:** 10.3389/fpsyt.2023.1212023

**Published:** 2023-05-12

**Authors:** Johannes Kirchebner, Elmar Habermeyer, Lena Machetanz, Anees Abrol

**Affiliations:** ^1^Department of Forensic Psychiatry, University Hospital of Psychiatry, University of Zurich, Zurich, Switzerland; ^2^Center for Translational Research in Neuroimaging and Data Science, Georgia State University, Atlanta, GA, United States

**Keywords:** machine learning (ML), forensic psychiatry, violence, offending, artificial intelligence

## Introduction

Machine learning (ML) as a modern form of statistical analysis is becoming more and more common in psychiatric research. The possibilities of ML to analyze complex relationships and potentially build predictive models seem particularly appealing regarding multifactorial yet poorly understood phenomena like violence and offending. This Research Topic aimed to gather evidence in this area to further promote the application of ML in forensic psychiatry. The contributing works comprise three original articles (Karystianis et al.; Popovic et al.; Yu et al.) and a systematic review (Parmigiani et al.).

The following two articles intended to identify affected individuals out of a sample through the use of ML algorithms. Yu et al. examined a population of male individuals with schizophrenia and were able to correctly identify aggressive patients with an AUC of 0.67. Here, the easy-to-survey variables of lower educational level, smoking, higher expression of positive symptoms, and social disability were the driving discriminating factors. Popovic et al. were able to distinguish pedophilic offenders from healthy comparisons with an accuracy of 75.5 through ML analysis, with alterations in the anterior cingulate cortex, the amygdala, and its cortical connections proving to be relevant predictor variables.

Karystianis et al. on the other hand used ML to extract information from nearly 500,000 police reports on domestic violence—a task that would be almost impossible to accomplish without the support of ML. Their findings showed a significant increase in registered injuries as well as reported mental illnesses in persons of interest. Furthermore, women appeared to show different types of injuries compared to men, even though the distribution of mental disorders was similar in both groups.

Last but not least, in their review, Parmigiani et al. examined studies attempting to predict violent behavior in clinical and forensic settings. They found that models with an AUC of up to over 0.8 were superior in their performance compared to established risk assessment tools. Nevertheless, the fact that these studies had entirely different populations and predictor variables has to be noted as a limitation.

## Conclusion

The collection in this Research Topic well demonstrates the potential of ML in the analysis of aggression and offending, including applications to data extraction (so-called mining), data analysis, and creation of predictive models to anticipate events relevant to forensic psychiatry. With ML becoming more widespread, an increase in studies with similar methodology can be well-expected. However, it is essential to ensure that the application and evaluation of the calculated models would be done on different patient populations—ideally international ones. Only then, generally valid predictors can be identified, and a robust generalizable model can be built and applied in clinical practice.

## Author contributions

All authors listed have made a substantial, direct, and intellectual contribution to the work and approved it for publication.

